# Risk Factors for Postoperative Morbidity, Suture Insufficiency, Re-Surgery and Mortality in Patients with Gastroduodenal Perforation

**DOI:** 10.3390/jcm12196300

**Published:** 2023-09-29

**Authors:** Julia Treuheit, Christian Krautz, Georg F. Weber, Robert Grützmann, Maximilian Brunner

**Affiliations:** Department of General and Visceral Surgery, Friedrich-Alexander-University Erlangen-Nürnberg (FAU), Krankenhausstraße 12, 91054 Erlangen, Germany; julia.treuheit@fau.de (J.T.); christian.krautz@uk-erlangen.de (C.K.); georg.weber@uk-erlangen.de (G.F.W.); robert.gruetzmann@uk-erlangen.de (R.G.)

**Keywords:** gastroduodenal perforation, surgery, risk factors, morbidity, mortality, re-surgery, suture insufficiency

## Abstract

(1) Background: The aim of the present study was to identify risk factors associated with postoperative morbidity, suture/anastomotic insufficiency, re-surgery, and mortality in patients undergoing surgery for gastroduodenal perforation. (2) Methods: A retrospective analysis of 273 adult patients who received surgical treatment for gastroduodenal perforation from January 2006 to June 2021 at the University Hospital Erlangen was performed. The patient demographics and preoperative, intraoperative, and postoperative parameters were collected and compared among the different outcome groups (in-hospital morbidity, suture/anastomotic insufficiency, re-surgery, and 90-day mortality). (3) Results: In-hospital morbidity, suture/anastomotic insufficiency, need for re-surgery, and 90-day mortality occurred in 71%, 10%, 26%, and 25% of patients, respectively. The independent risk factors for morbidity were a significantly reduced general condition, a lower preoperative hemoglobin level, and a higher preoperative creatinine level. The independent risk factors for suture/anastomotic insufficiency could be identified as an intake of preoperative steroids and a perforation localization in the proximal stomach or duodenum. The four parameters were independent risk factors for the need for re-surgery: a significantly reduced general condition, a perforation localization in the proximal stomach, a higher preoperative creatinine level, and a higher preoperative CRP level. An age over 66 years and a higher preoperative CRP level were independent risk factors for 90-day mortality. (4) Conclusions: Our study could identify relevant risk factors for the postoperative outcome of patients undergoing surgical treatment for gastroduodenal perforation. Patients exhibiting the identified risk factors should receive heightened attention in the postoperative period and may potentially benefit from personalized and tailored therapy.

## 1. Introduction

Gastroduodenal perforations are a common and highly critical emergency condition in visceral surgery, accounting for a significant proportion of surgical emergencies worldwide [1,2,3]. Peptic ulcers, in particular, significantly contribute to the incidence of gastroduodenal perforations, with studies reporting them as the leading cause in approximately 70–90% of cases. Moreover, gastroduodenal perforations can arise from various causes, including malignancies and iatrogenic interventions [4].

Studies reported high morbidity and mortality rates, varying from 30% to 70% and from 10% to 30%, respectively, reflecting the severity and complexity of these cases [1,5,6,7,8,9,10,11,12,13]. The morbidity and mortality rates associated with gastroduodenal perforations underline the urgent need for their prompt diagnosis and effective management.

Intra-abdominal sepsis, resulting from the leakage of gastric contents into the abdominal cavity, represents a significant challenge in the postoperative management of gastroduodenal perforations, as it is identified as a crucial factor contributing to a worse postoperative prognosis and can lead to complications such as abscess formation, septic shock, and multi-organ failure.

Surgical intervention remains the cornerstone of treatment for gastroduodenal perforations. The most common approach involves suturing the perforation, which can be successfully achieved in the majority of cases. However, gastric or duodenal resections are rarely required and are typically reserved for cases with extensive tissue damage or an associated malignancy [14,15].

The relevant frequency and poor prognosis associated with gastroduodenal perforations emphasize the necessity for continuously enhanced and personalized treatment approaches for affected patients [16]. Identifying postoperative risk factors that may adversely affect surgical outcomes can contribute to improving the quality of treatment by tailoring interventions and implementing individualized postoperative measures for high-risk patients.

The primary objective of the present study was to identify risk factors associated with the development of morbidity suture insufficiency, re-surgery, and mortality in patients undergoing surgical treatment for gastroduodenal perforation.

## 2. Materials and Methods

This retrospective analysis includes 273 consecutive patients (age ≥ 18 years) who underwent surgical management for acute gastroduodenal perforation at the Department of General and Visceral Surgery in University Hospital Erlangen between January 2006 and June 2021. Patients with one of the following criteria were excluded: (1) patients with age younger than 18 years; (2) patients without intraoperatively verified gastroduodenal perforation; (3) patients with esophageal perforation; (4) patients with conservatively or interventionally treated gastroduodenal perforation. 

Data about patients’ demographics, comorbidities, preoperative findings (blood results), surgical parameters, microbiological findings, and postoperative outcome were obtained and analyzed. A significantly reduced general condition at admission was defined as the presence of shock (heart rate > 100 beats per minute and systolic blood pressure < 100 mmHg) and/or the requirement of preoperative intensive care or reanimation. Morbidity was defined as any deviation from the normal postoperative course and classified according to the Clavien–Dindo classification [17]. Morbidity and mortality were assessed as in-hospital and 90-day parameters.

The main objective of this study was to determine the risk factors associated with in-hospital morbidity, suture/anastomotic insufficiency, re-surgery, and 90-day mortality following surgery for gastroduodenal perforation. Therefore, our study cohort was stratified into comparison groups based on these four parameters: no postoperative morbidity vs. postoperative morbidity, no suture/anastomotic insufficiency vs. suture/anastomotic insufficiency, no need for re-surgery vs. need for re-surgery, and no mortality vs. mortality.

This study was approved by the Ethics Committee of FAU Erlangen (23-194-Br).

### 2.1. Diagnostic and Therapeutic Algorithm of Patients with Gastroduodenal Perforation

Upon initial admission, all patients underwent a comprehensive blood test, which included a complete blood count, creatinine level, and inflammatory markers. Additionally, preoperative assessments such as abdominal ultrasound and abdominal X-ray were conducted. In cases where the diagnosis remained uncertain, an abdominal CT scan was performed (156 patients (56%)). The surgical procedure, including options such as suturing vs. resection, patch plastic or no patch plastic, and cut-out of the perforation vs. no cut-out of the perforation, was determined by the surgeon and documented in their surgical report. Suturing of perforation was performed using a single-layered single stitches techniques with PDS of 3 × 0 or 4 × 0. For additional patch plastic, an omental patch was preferred. Techniques did not differ between the open and the laparoscopic approaches. The decision to collect intraoperative samples for microbiological examination was again at the discretion of the surgeon.

### 2.2. Statistical Analysis

Statistical analyses were performed with SPSS Statistic (Version 28.0, IBM). Comparisons of ordinal and metric data were calculated by Mann–Whitney U test or Student’s *t*-test. For categorical data, the chi-square test was used. Statistical significance was set at *p* < 0.05. Multivariate analysis was performed with univariate analysis, which identified risk factors for the outcome parameters (morbidity, suture/anastomotic insufficiency, re-surgery, and mortality). As cutoffs for metric risk factors, the median was used. Independent risk factors in the multivariate analysis were included in analysis of risk factor score. 

## 3. Results

### 3.1. Patient Collective

A total of 273 patients, who underwent surgery for acute gastroduodenal perforation during the study period, were included. The median age of the patients was 66 years, and 47% of the patients were female. The most common cause of a gastroduodenal perforation was a peptic ulcera (64%), followed by iatrogenic interventions (12%) and malignancy (5%). Most of the gastroduodenal perforations were located in the distal stomach (65%), followed by in the duodenum (26%) and in the proximal stomach (9%). Overall, 90% of the gastroduodenal perforations were sutured, whereas 10% of the affected patients needed a partial or even a full gastrectomy. All demographic and perforation characteristics as well as the preoperative blood results, the surgical details, and the microbiological results are shown in Table 1. 

### 3.2. Outcome Parameter after Surgical Management of Gastroduodenal Perforations

In 194 patients (71%), postoperative morbidity occurred—including suture/anastomotic insufficiency in 10% and wound infection in 21%. In total, 41% of patients with postoperative complications suffered from a minor morbidity (Clavien–Dindo I and II), whereas 59% had a major morbidity (Clavien–Dindo III to V). Seventy-two patients (26%) required re-surgery. The median length of hospital stay was 11 days. Most patients were discharged to their home (93%). Fifty-nine (22%) and sixty-seven (25%) patients died during hospital stay and during the first 90 postoperative days, respectively. In 32 (15%) patients, readmission during the first 90 postoperative days was necessary (Table 2). 

### 3.3. Risk Factors for Postoperative In-Hospital Morbidity

In the univariate analysis, there were 15 risk factors, which were significantly associated with the occurrence of in-hospital morbidity: age > 66 years (*p* < 0.001), BMI > 25 kg/m^2^ (*p* = 0.002), presence of any comorbidity (*p* < 0.001), preoperative intake of steroids (*p* = 0.010), a significantly reduced preoperative general condition (*p* < 0.001), non-peptic ulcers–etiology of perforation (*p* < 0.001), location of perforation at the proximal stomach or duodenum (*p* = 0.037), a preoperative hemoglobin ≤ 12.4 g/dL (*p* < 0.001), a preoperative creatinine > 1.0 mg/dL (*p* < 0.001), a preoperative CRP level > 58 mg/L (*p* < 0.001), suturing of perforation with cut-out and without patch plastic (*p* < 0.001), an operative time > 94 min (*p* < 0.001), an intraoperative blood loss > 100 mL (*p* < 0.001), a positive intraoperative swab (*p* = 0.005), and a positive intraoperative mycotic swab (*p* = 0.010). The multivariate analysis revealed a significantly reduced preoperative general condition (HR 9.2 (1.1–78.1), *p* = 0.042), a preoperative hemoglobin ≤ 12.4 g/dL (HR 2.3 (1.0–5.1), *p* = 0.043), and a preoperative creatinine > 1.0 mg/dL (HR 2.3 (1.0–5.3), *p* = 0.044) as the independent risk factors for morbidity (Table 3a).

### 3.4. Risk Factors for Suture/Anastomotic Insufficiency

For the occurrence of a suture/anastomotic insufficiency, there were six associated parameters in the univariate analysis: BMI > 25 kg/m^2^ (*p* = 0.013), preoperative intake of steroids (*p* = 0.010), non-peptic ulcers–etiology of perforation (*p* = 0.010), location of perforation at the proximal stomach or duodenum (*p* < 0.001), a preoperative hemoglobin ≤ 12.4 g/dL (*p* = 0.015), and an intraoperative blood loss > 100 mL (*p* < 0.001). A preoperative intake of steroids (HR 3.3 (1.0-11.1), *p* = 0.049)) and location of perforation at the proximal stomach or duodenum (HR 14.0 (3.3–58.4), *p* < 0.001 and HR 3.9 (1.4–11.1), *p* = 0.011) remained significant risk factors in the multivariate analysis (Table 3a). 

### 3.5. Risk Factors for Re-Surgery

Univariately, 11 risk factors could be identified for the need for re-surgery: BMI > 25 kg/m^2^ (*p* < 0.001), presence of any comorbidity (*p* = 0.004), a significantly reduced preoperative general condition (*p* < 0.001), non-peptic ulcers–etiology of perforation (*p* < 0.001), location of perforation at the proximal stomach (*p* < 0.001), a preoperative hemoglobin ≤ 12.4 g/dL (*p* < 0.001), a preoperative creatinine > 1.0 mg/dL (*p* < 0.001), a preoperative CRP level > 58 mg/L (*p* < 0.001), need for resection procedure (*p* < 0.001), an operative time > 94 min (*p* = 0.003), and an intraoperative blood loss > 100 mL (*p* = 0.010). The multivariate analysis detected a significantly reduced preoperative general condition (HR 3.5 (1.5–8.2), *p* = 0.005)), location of perforation at the proximal stomach (HR 17.8 (3.4–93.1), *p* < 0.001), a preoperative creatinine > 1.0 mg/dL (HR 2.5 (1.1–5.6), *p* = 0.023), and a preoperative CRP level > 58 mg/L (HR 4.9 (2.1–11.5), *p* < 0.001) as independent risk factors for the need for re-surgery.

### 3.6. Risk Factors for 90-Day Mortality

There were 12 univariate risk factors for 90-day mortality: age > 66 years (*p* < 0.001), presence of any comorbidity (*p* < 0.001), a significantly reduced preoperative general condition (*p* < 0.001), non-peptic ulcers–etiology of perforation (*p* < 0.001), location of perforation at the duodenum (*p* = 0.045), a preoperative hemoglobin ≤ 12.4 g/dL (*p* < 0.001), a preoperative creatinine > 1.0 mg/dL (*p* < 0.001), a preoperative CRP level > 58 mg/L (*p* < 0.001), suturing of perforation with cut-out and without patch plastic and the need for resection procedure (*p* < 0.001), an intraoperative blood loss > 100 mL (*p* = 0.011), a positive intraoperative swab (*p* = 0.040), and a positive intraoperative mycotic swab (*p* = 0.023). Of these identified risk factors, only an age > 66 years (HR 2.3 (1.0–5.3), *p* = 0.042) and a preoperative CRP level > 58 mg/L (HR 4.2 (1.8–10.0), *p* < 0.001) were independent in the multivariate analysis (Table 3b).

### 3.7. Absolute Risk Values for Morbidity, Suture/Anastomotic Insufficiency, Re-Surgery, and 90-Day Mortality according to Presence of the Identified Independent Risk Factors

Table 4 shows the absolute risk values for in-hospital morbidity, suture/anastomotic insufficiency, re-surgery, and 90-day mortality according to the number of present, identified independent risk factors. 

## 4. Discussion

Gastroduodenal perforations represent a prevalent and extremely critical emergency situation requiring prompt diagnosis and effective management. Identifying risk factors for the most important outcome parameters could help to improve the quality of care by continuously enhancing and customizing treatment approaches specifically tailored for high-risk patients.

The demographic data of our study cohort were mostly similar to other studies reporting outcomes after surgical management for gastroduodenal perforations [5,6,7,8,9,10,11,12,13,14,15,16]. In our study, we selected in-hospital morbidity, suture/anastomotic insufficiency, reoperation, and 90-day mortality as the investigated outcome parameters, as these are the most relevant for affected patients.

The overall morbidity rate in our cohort was 71%, which is slightly higher compared to previous studies in the literature [5,6,7]. However, our cohort consisted of older patients compared to the mentioned studies, which could potentially explain the slight difference. Among the three identified risk factors for in-hospital morbidity, a significantly reduced preoperative general condition and higher preoperative creatinine levels were previously described in the literature [5,6]. In addition, we found that a lower preoperative hemoglobin level was an independent risk factor, which has not been reported in the literature until now. Nevertheless, a lower hemoglobin level is indicative of a poorer general condition in affected patients, and, therefore, an association with a higher risk for morbidity appears to be comprehensible. In contrast, other studies identified a higher age, a higher ASA score, open surgery, and a longer operation time as risk factors for morbidity [5,6]. However, these parameters were either not investigated in our study or could not be confirmed in our analysis.

The occurrence of postoperative suture/anastomotic insufficiency is a parameter that has not been extensively studied in previous research, resulting in the limited availability of comparable data. In our cohort, the rate of suture/anastomotic insufficiency was 10%, slightly higher than reported in the analysis by Wilhelmsen et al. [9]. However, Wilhelmsen et al. did not provide specific details on the surgical procedures that were performed, thus limiting the comparability of the findings. In our cohort, 10% of patients underwent gastric or duodenal resection with anastomosis, which is known to carry a higher risk of insufficiency compared to suturing a perforation. Regarding risk factors for the occurrence of suture/anastomotic insufficiency, we identified the preoperative use of steroids and the localization of the perforation in the proximal stomach or duodenum. Steroids are known to impede the healing process, making this an understandable association. Additionally, the proximity of the perforation to the proximal stomach or duodenum can be considered a risk factor, as perforations in these regions pose greater surgical challenges, and those in the proximal stomach often more frequently require resection procedures than those in the distal stomach or duodenum. To our knowledge, there are no comparable studies investigating risk factors specifically associated with this outcome parameter.

In our cohort, 26% of patients required reoperation. Compared to the aforementioned study by Wilhelmsen, the reoperation rate in our collective was again slightly higher [9]. However, we already acknowledged the limitations in comparability between the studies. Wilhelmsen et al. identified an open procedure as a risk factor for an increased reoperation rate in their analysis [9]. Moreover, a current meta-analysis of the perioperative outcomes of acute laparoscopic vs. the open repair of perforated gastroduodenal ulcers also showed no differences between the two techniques [15]. However, the laparoscopic approach was utilized in only a small proportion of cases (3%) in our study, which limits our ability to draw conclusions regarding this potential risk factor based on our data. Another study by Hasslager et al. reported male sex, in-hospital perforation, a high BMI, a high ASA physical status grade, shock on admission, surgical delay, and other comorbidities as risk factors for reoperation, with preadmission use of steroids and an age above 70 years identified as preventive factors [8]. However, apart from shock on admission, being defined as preoperative significantly reduced the general condition in our study, though none of these factors were identified as significant in our analysis. Nevertheless, our results identified three further risk factors that can be well-explained in connection with a higher reoperation rate. Elevated creatinine levels were already described as risk factors for increased morbidity, which, in turn, can explain a greater need for reoperation. The proximity of the perforation to the proximal stomach was associated with an increased rate of insufficiency, which often necessitates surgical revision. Additionally, higher C-reactive protein (CRP) levels likely indicate a more advanced inflammation caused by the perforation, which is also likely to be associated with an increased risk of intra-abdominal abscesses and subsequent complications as well as wound infections. These factors collectively contribute to the higher likelihood of requiring a repeat operation.

Lastly, we investigated 90-day mortality, which is a parameter that was extensively studied in the literature due to its significant relevance [5,6,7,8,9,10,11,12,13]. In our study, the mortality rate of 25% falls within the range of the previously published data (6–31%) [5,6,7,8,9,10,11,12,13]. Our analysis revealed a higher age and elevated C-reactive protein (CRP) levels as risk factors for mortality. An advanced age was consistently described as a prominent risk factor in the literature [5,12,13,18]. Regarding elevated CRP levels, they may serve as an indication of more severe inflammation, thus explaining the increased risk of mortality. Although CRP was not explicitly identified as a risk factor in previous studies, other research demonstrated that a delay in receiving appropriate treatment following perforation is associated with higher mortality rates [11,18]. Several other potential risk factors were identified in the literature, some of which could not be confirmed in our study, while others were not investigated. These include the need for reoperation, elevated creatinine levels, underweight, comorbidities, severe postoperative complications, the presence of cancer, delayed therapy after initial presentation, hypoalbuminemia, hyperbilirubinemia, the suspicion of liver cirrhosis, preoperative steroid use, preoperative shock, and the ASA score [5,9,10,11,12,13,18].

Consequently, the identified risk factors associated with surgical outcome parameters such as in-hospital morbidity, suture/anastomotic insufficiency, re-surgery, and 90-day mortality after surgery for gastroduodenal perforation can be utilized to implement early preventive measures in high-risk patients. Additionally, these risk factors can help in the early detection of complications through intensified postoperative monitoring, thus mitigating the severity of complications.

In contrast to most other studies that predominantly focus on peptic ulcer perforations, our study aimed to encompass all causes of gastroduodenal perforations, including those resulting from malignancies and traumas. By including these diverse causes, our data better represent the real-world scenario of gastroduodenal perforations. However, our study had some limitations that need to be acknowledged. Firstly, the retrospective nature of the study and its single-center design may introduce certain biases. Secondly, there were inherent interdependencies between certain parameters, such as the duration of surgery and the conversion rate, which can be influenced by the surgeons’ level of expertise.

## 5. Conclusions

In the context of surgical management for gastrointestinal perforations, accurate risk assessment plays a crucial role in identifying patients who are at a higher risk of experiencing unfavorable surgical outcomes. The implementation of risk classification can be valuable, as it enables targeted attention and personalized therapeutic approaches for high-risk patients during the postoperative phase.

## Figures and Tables

**Table 1:** Baseline characteristics for patients with gastroduodenal perforation.

Demographic data	Number	273
	Age (years), median (IQR)	66 (25)
	Gender, *n* (%)	
	Female	128 (47)
	Male	145 (53)
	ASA, *n* (%)	
	I	22 (8)
	II	63 (23)
	III	112 (41)
	IV	61 (22)
	V	2 (1)
	Unknown	13 (5)
	BMI (kg/m^2^), median (IQR)	24.5 (6.1)
	Comorbidity, *n* (%)	
	Hypertension	137 (50)
	Chronic renal insufficiency	51 (19)
	Diabetes	49 (18)
	Heart insufficiency	47 (17)
	Cardiovascular *	37 (14)
	COPD	31 (11)
	Active nicotine abuse, *n* (%)	109 (40)
	Steroid intake in the last three months before the onset of perforation, *n* (%)	21 (8)
Condition at presentation in the emergency room	Preoperative symptoms, *n* (%)	
	Pain	222 (81)
	Vomiting	69 (25)
	Weight loss	22 (8)
	Fever	12 (4)
	Preoperative general condition, *n* (%)	
	Well or slightly reduced	224 (82)
	Significantly reduced **	49 (18)
	Preoperative WBC (10^9^/L), median (IQR)	11.2 (8.2)
	Preoperative hemoglobin (g/dL), median (IQR)	12.4 (5.2)
	Preoperative creatinine (mg/dL), median (IQR)	1.0 (0.8)
	Preoperative CRP (mg/L), median (IQR)	58 (147)
Perforation characteristics	Etiology of perforation, *n* (%)	
	Peptic ulcera	175 (64)
	Others	98 (36)
	Malignancy	13 (5)
	Iatrogen	32 (12)
	Others (ideopathic, traumatic, consequence of another disease)	53 (19)
	Localization of perforation, *n* (%)	
	Stomach	202 (74)
	Proximal	24 (9)
	Distal	110 (40)
	Pyloric	68 (25)
	Duodenum	71 (26)
	Time from diagnosis to surgery (h), median (IQR)	3 (4)
Surgical details	Kind of procedure	
	Suturing	247 (90)
	Resection	26 (10)
	Surgical approach, *n* (%)	
	Open	270 (97)
	Laparoscopic	7 (3)
	Cut-out of perforation (only for suturing technique > *n* = 247), *n* (%)	70 (28)
	Additional patch plastic (only for suturing technique > *n* = 247), *n* (%)	160 (64)
	Operative time (min), median (IQR)	95 (57)
	Intraoperative blood loss (ml), median (IQR)	100 (250)
Microbiology	Intraoperative swab positive, *n* (%)	174 (64)
	Intraoperative swab positive for fungi, *n* (%)	110 (40)

IQR = interquartile range; ASA = American Society of Anesthesiologists score; BMI = body mass index; COPD = chronic obstructive pulmonary disease; WBC = white blood cell count; CRP = C-reactive protein. * except heart insufficiency; ** defined as the presence of shock (heart rate > 100 beats per minute and systolic blood pressure < 100 mmHg) and/or the requirement of preoperative intensive care or reanimation.

**Table 2:** Outcome parameters after surgery for gastroduodenal perforation.

Time after Surgery	Outcome Parameter	Number of Cases (%)
In-hospital	Clavien–Dindo, *n* (%)	
	0	79 (29)
	I	37 (14)
	II	42 (15)
	III	23 (8)
	IV	33 (12)
	V (Mortality)	59 (22)
	Suture/anastomotic insufficiency, *n* (%)	27 (10)
	Wound infection, *n* (%)	56 (21)
	Re-surgery, *n* (%)	72 (26)
	Length of postoperative hospital stay (in days), median (IQR)	11 (19)
After discharge	Discharge to (*n* = 214) *, *n* (%)	
	Home	199 (93)
	Other medical department	9 (4)
	Palliative care	5 (2)
	Rehabilitation	1 (0)
	Readmission (during 90 days postoperative) (*n* = 214) *, *n* (%)	32 (15)
	Morbidity (during 90 days postoperative), *n* (%)	202 (74)
	Mortality (during 90 days postoperative), *n* (%)	67 (25)

IQR = interquartile range. * excluding patients with postoperative in-hospital mortality.

**Table 3:** (**a**) Pre- and intraoperative risk factors for outcome parameters (in-hospital morbidity and suture/anastomotic insufficiency) in patients after surgery for gastroduodenal perforation. (**b**) Pre- and intraoperative risk factors for outcome parameters (re-surgery and 90-day mortality) in patients after surgery for gastroduodenal perforation.

(a)						
	In-Hospital Morbidity (*n* = 194)			Suture/Anastomotic Insufficiency (*n* = 27)		
	Multivariate			Multivariate		
	HR	95% CI	*p*	HR	95% CI	*p*
**Age (** **≤66 vs. >66 years)**	1.7	0.7–4.0	0.203	-	-	-
**BMI (** **≤25 vs. >25 kg/m^2^)**	1.4	0.6–3.2	0.434	1.7	0.6–4.4	0.286
**Any comorbidity (no vs. yes)**	0.8	0.3–2.0	0.664	-	-	-
**Preoperative steroids (no vs. yes)**	5.3	0.6–47.3	0.136	**3.3**	**1.0–11.1**	**0.049**
**Significantly reduced preoperative general condition (no vs. yes)**	**9.2**	**1.1–78.1**	**0.042**	-	-	-
**Etiology of perforation (peptic ulcera vs. others)**	2.4	0.9–6.4	0.087	0.9	0.3–2.8	0.923
**Localization of perforation**						
** - proximal stomach**	1.0			1.0		
** - distal stomach including pyloric**	0.6	0.1–5.0	0.674	**0.1**	**0.0–0.3**	**<0.001**
** - duodenum**	1.1	0.1–8.6	0.949	0.3	0.1–1.1	0.064
**Preoperative hemoglobin (≤12.4 vs. >12.4 g/dL)**	**0.4**	**0.2–1.0**	**0.043**	0.3	0.1–1.0	0.051
**Preoperative creatinine (≤1.0 vs. >1.0 mg/dL)**	**2.3**	**1.0–5.3**	**0.044**	-	-	-
**Preoperative CRP (≤58 vs. >58 mg/L)**	1.8	0.8–3.9	0.145	-	-	-
**Kind of surgery**				-	-	-
** - suturing with CU and PP**	1.0					
** - suturing with CU without PP**	3.1	0.6–15.0	0.166			
** - suturing without CU with PP**	1.8	0.7–4.7	0.260			
** - suturing without CU and PP**	1.8	0.5–7.1	0.402			
** - resection**	1.7	0.3–9.3	0.564			
**Operative time (≤94 vs. >94 min)**	1.7	0.8–3.8	0.188	**-**	**-**	**-**
**Intraoperative blood loss (≤100 vs. >100 mL)**	1.5	0.7–3.5	0.289	2.2	0.8–6.0	0.128
**Positive intraoperative swab (no vs. yes)**	1.2	0.5–3.1	0.672	-	-	-
**Positive intraoperative mycotic swab (no vs. yes)**	2.1	0.8–6.4	0.127	-	-	-
(**b**)						
	**Re-Surgery** **(*n* = 72)**			**90-Day Mortality** **(*n* = 67)**		
	**Multivariate**			**Multivariate**		
	**HR**	**95% CI**	**p**	**HR**	**95% CI**	**p**
**Age (** **≤66 vs. >66 years)**	-	-	-	**2.3**	**1.0–5.3**	**0.042**
**BMI (** **≤25 vs. > 25kg/m^2^)**	1.9	0.9–4.1	0.100	-	-	-
**Any comorbidity (no vs. yes)**	0.9	0.3–2.1	0.724	1.9	0.7–4.9	0.184
**Significantly reduced preoperative general condition (no vs. yes)**	**3.5**	**1.5–8.2**	**0.005**	2.1	0.9–4.9	0.079
**Etiology of perforation (peptic ulcera vs. others)**	1.2	0.5–2.9	0.680	1.5	0.6–3.7	0.332
**Localization of perforation**						
** - proximal stomach**	1.0			1.0		
** - distal stomach including pyloric**	**0.1**	**0.0–0.3**	**<0.001**	1.2	0.3–4.9	0.795
** - duodenum**	**0.1**	**0.0–0.5**	**0.004**	2.7	0.6–12.2	0.188
**Preoperative hemoglobin (≤12.4 vs. > 12.4g/dL)**	0.6	0.3–1.3	0.180	0.5	0.2–1.1	0.073
**Preoperative creatinine (≤1.0 vs. > 1.0mg/dL)**	**2.5**	**1.1–5.6**	**0.023**	2.0	0.9–4.5	0.077
**Preoperative CRP (≤58 vs. > 58mg/L)**	**4.9**	**2.1–11.5**	**<0.001**	**4.2**	**1.8–10.0**	**<0.001**
**Kind of surgery**						
** - suturing with CU and PP**	1.0			1.0		
** - suturing with CU without PP**	1.0	0.2–5.1	0.997	2.8	0.6–13.1	0.195
** - suturing without CU with PP**	1.4	0.4–4.9	0.578	1.1	0.3–4.2	0.835
** - suturing without CU and PP**	0.8	0.2–3.1	0.698	2.0	0.5–7.9	0.342
** - resection**	2.9	0.6–13.6	0.175	2.9	0.6–13.6	0.181
**Operative time (≤94 vs. >94 min)**	1.3	0.6–2.9	0.585		-	-
**Intraoperative blood loss (≤100 vs. >100 mL)**	0.9	0.4–2.0	0.853	1.2	0.6–2.7	0.575
**Positive intraoperative swab (no vs. yes)**	-	-	-	0.9	0.3–2.6	0.860
**Positive intraoperative mycotic swab (no vs. yes)**	-	-	-	1.6	0.6–4.2	0.318

BMI = body mass index; WBC = white blood cell count; CRP = C-reactive protein; CU = cut-out; PP = patch plastic.

**Table 4:** Risk for morbidity, suture/anastomotic insufficiency, re-surgery, and 90-day mortality depending on the number of identified independent risk factors.

Number of Risk Factors	In-Hospital Morbidity *	Suture/Anastomotic Insufficiency **	Re-Surgery ***	90-Day Mortality ****
	risk	risk	risk	risk
**0**	43.2%	5.7%	8.8%	1.2%
**1**	75.3%	31.7%	14.1%	25.4%
**2**	87.3%	50.0%	44.3%	51.5%
**3**	100%		70.0%	
**4**			100%	

* Three independent risk factors for morbidity: significantly reduced preoperative general condition, preop. hemoglobin ≤ 12.4 g/dL, and preoperative creatinine > 1.0 mg/dL. ** two independent risk factors for suture/anastomotic insufficiency: preoperative steroids and localization of perforation in proximal stomach or duodenum. *** four independent risk factors for re-surgery: significantly reduced preoperative general condition, localization of perforation in proximal stomach, preoperative creatinine > 1.0 mg/dL, and preoperative CRP > 58 mg/L. **** two independent risk factors for 90-day mortality: age > 66 years and preoperative CRP > 58 mg/L.

## Data Availability

All data generated or analyzed during this study are included in this published article.
